# Misoprostol Alone or in Combination with Methotrexate for Termination of Pregnancy at First Trimester

**Published:** 2010

**Authors:** Fatemeh Vahid Roudsari, Sedigheh Ayati, Nafiseh Saghafy, Mohamadtaghi Shakeri

**Affiliations:** a *Department of Obstetrics and Gynecology, Mashhad University of Medical Sciences, Women’s Health Research Center, Ghaem Hospital, Mashhad, Iran.*; b *Department of Medicosocial, Biostatics Unit, Mashhad University of Medical Sciences, Ghaem Hospital, Mashhad, Iran.*

**Keywords:** Missed abortion, Misoprostol, Methotrexate, Early medical abortion, Pregnancy termination

## Abstract

Abortion is an important problem in obstetrics throughout the world. The common and standard method for pregnancy termination at first trimester is surgery (curettage). Nowadays, an effective method of pregnancy termination at first trimester is medical treatments. The aim of this study is to compare misoprostol alone or in combination with methotrexate for pregnancy termination at first trimester. This study is a randomized clinical trial. A total of 200 pregnant women at first trimester were randomizedly divided into two groups for termination of pregnancy. The first group received 800 μg vaginal misoprostol. If conceptus residual remained, the same dose of misoprostol was repeated. The second group received 50 mg/m² intramuscular methotrexate, and then 800 μg vaginal misoprostol was administered after 72 h. If conceptus residual remained, the same dose of misoprostol was repeated after 24 h. Abdominal ultrasonography was performed at seventh day for both groups. Should conceptus residual remained or if pregnancy continued, curettage was performed. The results were analyzed statistically in terms of chi-square, and student’s t-test, using the SPSS software. A P-value equal or smaller than 0.05, was considered statistically significant. In this study, 83% of the first group and 81% of the second group had successful abortion. There was a significant correlation between the dose of misoprostol and abortion (P = 0.001) and between type of pregnancy and need for curettage (P < 0.000) in both groups, but there was no significant correlation between gestational age and the numberof doses administered (P = 0.932).In conclusion it seems that pregnancy termination by misoprostol alone or in combination with methotrexate is a safe and cost-effective method.

## Introduction

The management of normal pregnancy without any side-effects, with the purpose of saving the life of mother and fetus, is one of the important duties of physician. However, but sometimes despite the desire of mother for continuing pregnancy, the physician should perform abortion, for example in cases that mother is complicated by pulmonary hypertension or a fetus that has severe anomaly (including anencephaly or Down’s syndrome) (1).

In the recent decades, medical pregnancy termination methods have become a suitable replacement for surgical methods. Three medications for early medical abortion have been studied: the antiprogestin mifepristone, the antimetabolite methotrexate, and the prostaglandin misoprostol. These agents cause abortion by increasing uterine contractility, either by stimulating the myometrium directly (misoprostol) or by reversing the progesterone-induced inhibition of contraction ( mifepristone and methotrexate) (1).

Methotrexate has been used safely and successfully to treat unruptured ectopic pregnancy (2-4). It has long been known that methotrexate is cytotoxic to proliferative trophoblastic tissue. Hence, so it has been used to treat malignant trophoblastic and other epithelial tumors (5). In addition, misoprostol has been approved by the Food and Drug Administration for the prevention of gastric ulcer disease. Creinin and Vittinghoff used misoprostol either alone or in combination with methotrexate up to 56 days from the last menstrual period (6). They reported that 90 percent of their patients had complete abortion. Lamakov and coworkers have reported a success rate of 96% in termination of pregnancies which were less than 7 weeks, by the use of misoprostol in combination with methotrexate. They concluded that these two drugs are safe and effective in medical abortion and suggested them to obstetricians (7). In another study performed in 2004 at South Africa, 104 patients were compared for the efficacy of medical abortion and expectant management (8). The success rate of treatment with misoprostol was found to be 87% and in the expectant management 29%, in this study. 

The aim of this study is to compare misoprostol alone or in combination with methotrexate for termination of pregnancy at first trimester.

## Experimental

The present study is a randomized clinical trial. The study population included 200 pregnant women at their first trimester (gestational age <13 weeks), who had referred to teaching hospitals related to Mashhad University of Medical Sciences during 2004-2007 for pregnancy termination.

The inclusion criteria of this study were:

a) Complete awareness of patients from both medical and surgical methods for pregnancy termination and its side-effects.

b) An intrauterine pregnancy < 13 weeks on the basis of last menstruation period or abdominal ultrasonography.

c) Specific reasons for pregnancy termination (missed abortion, blighted ovum and therapeutic abortion).

The exclusion criteria of this study were hypersensitivity to misoprostol, severe anemia (hemoglubin < 10g/dL), coagulopathy disorders or the use of anticoagulant drugs, acute liver and adrenal disease, cardiovascular diseases, uncontrolled seizure, and the use of corticosteroids.

The patients were randomisedly divided into two groups of 100 patients. The first group was treated with vaginal misoprostol alone and the second group with misoprostol and methotrexate. Misoprostol (Cytotec®) was manufactured by Pfizer (Madrid) and methotrexate by the Ebeve Pharama (Austria). The study’s protocol, the side-effects and benefits of medical abortion, the visit schedule and informed consent were reviewed with each subject. Informed consent included both the patient and her husband’s consent to perform an elective abortion with the understanding that there would be a surgical abortion if the medical abortion failed. All the women were asked to stop taking vitamin supplements containing folate. The estimated gestational age was based on the last menstrual period and abdominal ultrasonography. Baseline values of complete blood count, platelet count, liver function tests and serum creatinine were measured and if there was any abnormality in laboratory tests, the patients were excluded from the study. Moreover, both methods had been approved by the Ethics Committee of Mashhad University of Medical Sciences. A questionnaire was completed for each patient, including age, gestational age, gravidity, type of pregnancy, doses of misoprostol administration, need for curettage and drug side-effects. 

**Table 1:** Demographic characteristics of the studied patients

**Parameters **	**Group1 **	**Group 2 **	**P-value **
Mean age	27 ± 5	27 ± 2	P = 0.1
Mean number of pregnancy	2 ± 1	2 ± 1.6	P = 0.1
Mean gestational age	10 ± 2	9 ± 1	P > 0.1

The fir**s**t group received 800 μg vaginal misoprostol in the posterior fornix of vagina by clinician without any additional intervention. The patients were controlled for 4 h and if no complication appeared, they were discharged. 

If conceptus expulsion did not occur based on patient's report, they received another 800 μg vaginal doses of misoprostol. After one week, abdominal ultrasonography was performed and curettage was carried out if residue remained or if pregnancy continued. Moreover, BG + Rh and liver and renal tests were determined at first visit for both groups. 

The second group received an intramuscular administration of 50 mg/m² of methotrexate at first visit and returned after 72 h (second visit) for vaginal administration of 800 μg misoprostol. At the third visit (24 h later), if expulsion did not occur, 800 μg of vaginal misoprostol was again inserted in the posterior fornix of vagina. After one week, abdominal ultrasonography was performed and curettage was conducted if residue remained or if pregnancy continued. 

The assessed outcomes were including abortion success, duration of vaginal bleeding, and side-effects. A successful abortion was defined as complete abortion, confirmed by ultrasound examination without the requirement for a surgical procedure. 

Following the collection of study data, they were analyzed statistically (chi-square, and student's t-test), using SPSS software. A P-value equal to or less than 0.05 was considered statistically significant. Quantitative variables were calculated as mean ± SD. 

## Results

In this study, there was no significant correlation between the two groups, as they were compared in terms of age, number of pregnancy and gestational age (Table 1). 

In the first group, among 83 patients that had abortion, 55 (62%) cases had successful abortion with one dose of misoprostol and 28 (34%) with two doses (chi-square = 8.78, P < 0.001). 

In the second group, among 81 patients that had abortion, 60 (79%) cases had successful abortion with one dose of misoprostol and 21 (40%) with two doses (chi-square =18.71, P < 0.001) (Table 2). 

**Table 2:** Correlation between the number of drug administration and need for curettage in both groups

**Need to curettage **	**First group**		**Second group**		**P-value**
	**Yes**	**No**	**Yes**	**No**	
First dose	3 (5.2%)	55 (62%)	0 (0%)	60 (79%)	0.074
Second dose	14 (33.3%)	28 (34%)	19 (47.5%)	21 (40%)	0.191
Total	17 (38.5%)	83 (96%)	19 (19%)	81 (81%)	0.713
The results of Statistical tests	P = 0.001		P = 0.001		

 There was no significant correlation between dose of misoprostol and the need to curettage in both groups (chi-square = 1.19, P = 0.275). 

In terms of gestational age, patients were divided into two groups: gestational age of ≤9 weeks and gestational age of > 9 weeks. In both groups, there was no significant correlation between the gestational age and the dose of misoprostol (chi-square = 0.007 , P = 0.934 in the first group and chi-square = 0.585 , P = 0.44 in the second group respectively) (Table 3). 

**Table 3:** Correlation between the gestational age and the number of doses of misoprostol in both groups.

**Groups **	**First group**		**Second group**		**P-value**
	**First dose **	**Second dose **	**First dose **	**Second dose **	
Gestational age of ≤ 9 week	23 (57.5%)	17 (42.5%)	16 (66.7%)	8 (33.3%)	0.467
Gestational age of > 9 week	35 (41.7%)	25 (58.3%)	44 (57.9%)	32 (42.1%)	0.959
Total	58 (58%)	42 (42%)	60 (60%)	40 (40%)	0.774
Results of statistical tests	P = 0.934		P = 0.444		

In the first group, there was a significant correlation between the gestational age and the need to curettage (chi-square = 4.264, P = 0.039). However in the second group, there was no significant correlation between these two variables (chi- square = 2.335, P = 0.127) (Table 4). 

**Table 4:** Correlation between the gestational age and the need for curettage in both groups

** Groups ** ** (need for curettage) **	**First group**		**Second group**		**P-value**
	**Yes**	**No**	**Yes**	**No**	
Gestational age of ≤ 9 week	3 (7.5%)	37 (92.5%)	2 (8.3%)	22 (91.7%)	0.904
Gestational age of > 9 week	14 (23.3%)	46 (76.7%)	17 (22.4%)	59 (77.6%)	0.894
Total	17 (17%)	83 (83%)	19 (19%)	81 (81%)	0.713
Results of statistical tests	P = 0.039		P = 0.127		

In terms of the type of pregnancy, the patients were divided into two groups: (a) missed abortion

and (b) blighted ovum or therapeutic abortion. In the first group, there was no significant correlation between the type of pregnancy and the dose of misoprostol (chi-square = 0.618, P = 0.439) and in the second group, there was a significant correlation between these two variables (chi-square = 15.425, P < 0.001). 

In both groups, there was a significant correlation between the type of pregnancy and curettage (chi-square = 3.83 and P = 0.051in the first group, and chi-square = 4.258 and P = 0.038 in the second group). 

In the present study, no important side-effects were observed with the use of misoprostol or methotrexate. 

## Discussion

The clinical experience of this study suggests that early pregnancy termination with misoprostol alone or the combination of low-doses of methotrexate and vaginal misoprostol is both safe and effective. Although the toxicity of methotrexate therapy for cancer is extensive, the doses used for cancer treatment are much larger than those in this study. 

In the present study, misoprostol alone was effective in medical abortion in 83% of the subjects. There have been relatively few trials, which have evaluated the use of misoprostol alone for early pregnancy termination (9). In a clinical study using with 800 μg vaginal misoprostol, there were 47% complete abortion, 27% incomlplete abortion and 27% ongoing pregnancy (6). Koopersmith and Mishell have reported an extent of 61% complete abortion with doses of 200 and 400 μg misoprostol alone, as did Bugalho et al. who showed success rates of 66% and 46% with 400 and 200μg misoprostol alone, respectively (10, 11). In a large series, Carbonell et al. found a high success rate of 94% by using repeated doses of misoprostol and reported this method would increase the efficacy and help to progress incomplete abortion to complete abortion (12). In a study performed in Florida in 2004, vaginal misoprostol was administered to 41 pregnant women for first trimester abortion, and the rate of success obtained was 78% (13). This rate of success observed is lower than our study, which could be due to the fewer numbers of studied patients in their study. 

The combination of misoprostol and methotrexate resulted in a complete and safe termination of pregnancy, as demonstrated previously (14-17). In the present study, the rate of success with this method was 81%. In the study performed in 2001 by Borgatta et al., methotrexate in combination with vaginal misoprostol was administered in pregnancy with a gestational age of < 49 days, and 84% of cases was found to have complete abortion (18). In the present study, the rate of success with this method in cases with a gestational age of < 9 weeks was 83% and for patients with a gestational age of ≤ 7 weeks was 100%. Creinin et al, in USA, evaluated the efficacy of misoprostol and methotrexate in 300 pregnant women with a maximum gestational age of 56 days. They reported that 90% of women with a gestational age of 49 days and 81.6% with a gestational age of 50-56 days had complete abortion (19). In our study, following the administration of misoprostol and methotrexate in women with a gestational age of 9 weeks, 83% had complete abortion and following the administration of misoprostol alone in the same gestational age, 92.5% had complete abortion. The differences obtained between these two studies are most probably due to the variation in the type of patients. All the studied patients by Creinin et al. had live fetus, but in the present study, the patients with missed abortion and blighted ovum also entered the study. In the performed study by Beucher in 2004, vaginal misoprostol (800 μg) was administered for 102 pregnant women at first trimester. They showed that 72 cases had missed abortion and 30 patients threatened abortion. The rate of success was reported to be 78.4% (20). Our study is statistically similar to the study performed by Beucher et al. in 2004 which could be due to the similar number and type of patients in both studies. Previous studies using methotrexate followed 3-7 days later by vaginally administered misoprostol, had success rates of 72% to 98% (21). Misoprostol had been reported to be more effective, when given 7 days instead of 3, 4 or 5 days after administration of methotrexate (16). In the study performed by Jahangir et al. at 2005, the efficacy of misoprostol for termination of pregnancy in missed abortion was evaluated. They suggested this method as an effective policy without side-effects, and a good replacement for surgical methods (22). 

Misoprostol alone or in combination with methotrexate could be an acceptable method for the first trimester abortion. Since the rate of success is similar in both methods, it is possible to eliminate the administration of methotrexate which is a cytotoxic drug with many potential side-effects. Of course, it should be mentioned that side-effects of methotrexate appear in large doses for chemotherapy and for our patients that have received low doses (50 mg/m²), no important side-effects was observed. For administration of methotrexate, it is necessary to perform liver and renal tests and CBC ,which would all bear extra costs. However, with the use of misoprostol alone, the cost of medical abortion and the number of visits are effectively decreased and there is no need for intramuscular injection. 

In conclusion, the use of methotrexate in low dose in combination with misoprostol or alone for the first trimester termination are both safe and effective methods without important side-effects. These two methods can be used alternatively, but the results of the present study suggest misoprostol alone is easier. This is due to the fact that the number of visits is decreased, there is no need for intramuscular injection and possible side-effects of methotrexate that is a cytotoxic drug are eliminated. Most of the patients expressed their consent of this method and suggested it to other patients.
